# Response of the soil hydrothermal environment and cotton yield to different irrigation quotas under biodegradable mulch film in oasis cotton fields: a three-year study

**DOI:** 10.3389/fpls.2025.1521635

**Published:** 2025-03-21

**Authors:** Hao Zhang, Dong Wang, Xun Zhang, Yifan Wang, Haijun Liu, Qiuxiang Tang, Tao Lin

**Affiliations:** ^1^ College of Agriculture, Xinjiang Agricultural University, Urumqi, China; ^2^ Xinjiang Jinfengyuan Seed Industry Co., LTD., Xinjiang, China; ^3^ Xinjiang Cotton Technology Innovation Center/Xinjiang Key Laboratory of Cotton Genetic Improvement and Intelligent Production/National Cotton Engineering Technology Research Center, Cotton Research Institute of Xinjiang Uyghur Autonomous Region Academy of Agricultural Sciences, Wulumuqi, Xinjiang, China

**Keywords:** biodegradable mulch film, irrigation quota, accumulative soil temperature, soil water content, cotton yield

## Abstract

**Introduction:**

Polyethylene mulch film (PE) is a key agricultural practice for enhancing crop production and income in water-scarce regions. However, the complete recycling of PE remains challenging, resulting in the persistence of residual film fragments in the soil, which compromises soil structure and negatively impacts crop growth and yield potential. Although biodegradable mulch film (BEMF) is considered a promising alternative, the underlying mechanisms governing its regulation of soil water and thermal dynamics, as well as its subsequent impacts on crop productivity, are yet to be fully elucidated.

**Methods:**

Therefore, a comprehensive understanding of how BEMF influences soil water dynamics, thermal regimes, and crop growth and development is crucial for assessing its ecological adaptability. In this study field plot experiments were carried out over three consecutive growing seasons (2021 - 2023) under three irrigation quotas: W1 (63.6% crop evapotranspiration [ETc], 315 mm), W2 (81.8% ETc, 405 mm), and W3 (100% ETc, 495 mm).

**Results:**

This study systematically evaluated the impacts of PE and biodegradable mulch films (BEMF: B1 and B2) on soil hydrothermal dynamics, cotton photosynthetic productivity, and water use efficiency under varying irrigation quotas. Furthermore, the economic and ecological benefits of cotton fields under these treatments were analyzed. The findings revealed that PE left residual film fragments of 12.95 kg·ha^-1^ in the soil after mechanical recovery, while BEMF exhibited no such residue accumulation. However, BEMF reduced soil effective temperature by 100 - 111°C and soil water content (SWC) by 2.82 - 9.42% compared to PE. These adverse effects under BEMF significantly impaired cotton net photosynthetic rate (Pn) and photosynthetic product accumulation. Specifically, BEMF decreased cotton net Pn by 8.42 - 18.09%, photosynthetic product accumulation by 10.74 - 26.41%, and yield by 651 - 1079 kg·ha^-1^ relative to PE, particularly under the W1 irrigation level. Increasing the irrigation quota mitigated soil water and heat deficits, enhanced cotton net Pn and photosynthetic productivity, boosted yield by 1.76 - 31.72%, and increased economic income by 552 - 12,423 CNY·ha^-1^.

**Discussion:**

In summary, this study provides a new ecological regional adaptation scheme for BEFM, highlighting that under conventional conditions, BEFM cannot fully substitute the yield advantages of PEFM. Nevertheless, the application of an additional 90 mm of irrigation water effectively mitigates the yield and economic losses associated with BEMF while eliminating the risk of residual film fragment accumulation in the soil. These findings offer valuable insights for advancing the green and sustainable management of agricultural ecosystems.

## Highlights

1. Biodegradable mulch film reduces soil temperature and water content, decreasing cotton yield compared to polyethylene mulch film.
2. Increasing irrigation quota compensates for water and heat loss, promoting cotton growth and yield under biodegradable mulch film.
3. Based on comprehensive consideration of the economic and ecological benefits of cotton fields, an additional 90 mm of irrigation water can offset the loss of cotton yield and economic benefits of biodegradable mulch film while preventing mulch film fragments from remaining in the soil.

## 1 Introduction

Mulching causes warming and moisture preservation and improves crop yield and water use efficiency by inhibiting long-wave surface radiation and blocking the transfer of water vapor between the soil surface and the atmosphere (Gu et al., 2016; Sun et al., 2020; Wang et al., 2015; Zhao et al., 2023). As such, its use is a key means of promoting agricultural production and increasing income in areas with water shortages (Liu et al., 2022) For example, in the oasis cotton region of Xinjiang, the application of plastic film mulching technology increased cotton yield by 36.7% (Yan et al., 2010). However, due to a lack of awareness, plastic film is not effectively recovered after use, leading to the continuous accumulation of a large amount of plastic film fragments in agricultural soil, which has caused irreparable negative impacts on the soil’s physicochemical properties and crop yields (He et al., 2018; Yang L. et al., 2023; Yang X. et al., 2023). Zhang et al. (2020)found through a global meta-analysis that for every additional 100 kg·ha-1 of plastic film residue, the soil water infiltration rate decreases by 8%, nutrient content declines by 0.8% - 5%, root weight of crops decreases by 5%, and yields reduce by 3%. Over time, the yield-reducing effects of plastic film residue will outweigh the yield-increasing effects of plastic film coverage (Gu et al., 2024). Therefore, effective alternative methods should be implemented immediately to suppress further increases in plastic film residue while ensuring the benefits of plastic film coverage, in order to maintain the sustainable development of agriculture.

Biodegradable mulch film (BEMF) is directly degraded into CO_2_ and H_2_O in the middle and late stages of crop growth (Serrano-Ruiz et al., 2021; Sintim et al., 2019). It can play a similar role in warming and moisture preservation as PE (Tofanelli and Wortman, 2020; Wang et al., 2019) and can avoid irreversible residual film accumulation pollution (Huang et al., 2023). Therefore, it is considered a substitute for PE and has been verified as such in many crops. However, the stability of the BEMF degradation cycle is affected by material and meteorological factors, often leading to fluctuations in production and efficiency that, to some extent, affect the prospects for the application of this technology. In Spain and Portugal, studies by Moreno and Moreno (2008) and Costa et al. (2014) indicate that Biodegradable Enhanced Mulch Film (BEMF) possesses all the functions of Plastic Enhanced Mulch Film (PE) and does not reduce the yield and quality of tomatoes and strawberries after degradation. Research by Adamczewska-Sowińska and Turczuk (2018) and Cozzolino et al. (2023) in Poland and Italy shows that BEMF may degrade and break down in the later stages of crop growth, resulting in losses of moisture and temperature, yet it does not have a negative impact on the yield of crops such as tomatoes and melons. In southern China (Yang C. et al., 2023), BEMF with an appropriate degradation rate can prevent declines in the yield and quality of potatoes caused by excessive moisture accumulation and high temperatures. However, Graf et al. (2024) indicates that under the temperate climate conditions in the UK, the current BEMF cannot replace PE to enhance corn yields. In the northwestern regions of China, crops such as cotton (Wang et al., 2019), maize (Meng et al., 2022), and processing tomatoes (Jia et al., 2020) experience yield performance that is inferior to PE due to moisture and temperature losses caused by the degradation and breakdown of BEMF. Therefore, finding a method to prevent yield loss under BEMF coverage is essential for its continued promotion and application.

Changes in soil moisture and temperature also result in crop yield differences under BEMF. Gu et al. (2017) and Tofanelli and Wortman (2020) found that after BEMF degraded, its performance in maintaining soil temperature and water storage significantly decreased compared to PE but had a slightly lower soil temperature, which did not significantly affect crop growth or yield formation. However, Meng et al. (2022); Liu et al. (2022), and Jia et al. (2020) found that the yield of crops, such as corn, cotton, and processed tomatoes, significantly decreased due to soil water loss and thermal factors caused by BEMF.

So far, PE has greatly facilitated the development of modern agriculture (Liu et al., 2022), especially in arid and semi-arid regions, which account for approximately 45% of the Earth’s land area (Volkman et al., 2010). In these areas, the biggest challenge faced by biodegradable film applications compared to PE is their relatively poor warming and moisture retention effects, which may fail to meet the normal requirements of crops and could impact yield formation and the benefits to growers. Therefore, we hypothesize that appropriately adjusting irrigation quotas can offset the losses of moisture and temperature under biodegradable film coverage, thus increasing crop yield without reducing the benefits to growers, while avoiding the increase of plastic film fragments in the farmland. To validate this hypothesis, we conducted a three-year field experiment using cotton as the test crop in the typical arid and semi-arid region of the Xinjiang Oasis. The objectives of the study are: (1) To investigate whether increasing irrigation quotas will have a positive effect on cotton yield under BEMF coverage; (2) To analyze the changes in soil temperature and moisture content under different irrigation quotas with BEMF coverage; (3) To elucidate the impact of changes in soil temperature and moisture content on the photosynthetic production process and yield of cotton; (4) To clarify the mechanisms of yield variation in cotton under different irrigation quotas.

## 2 Materials and methods

### 2.1 Overview of the test area

**Figure 1:**
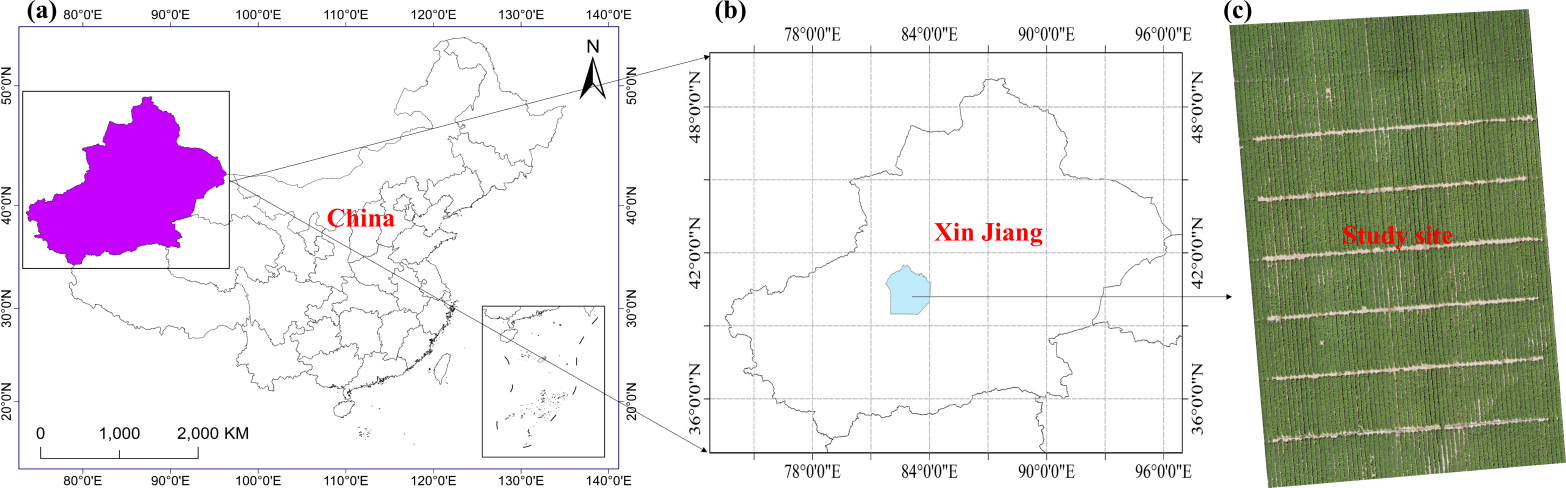
Xinjiang, located in Northwest China **(a)**, is characterized by a desert climate. The study site belongs to an oasis agroecosystem in South Xinjiang. **(b, c)** Experiments were conducted in a cotton-planting field (41°17′ N, 82°42′E) near Shaya County.

**Figure 2:**
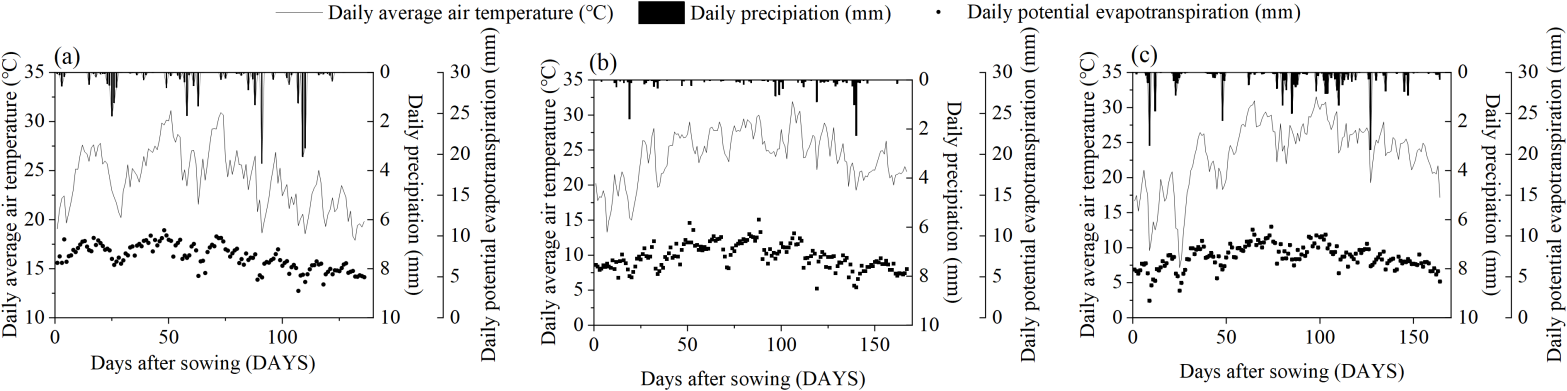
Monthly daily average air temperature, daily precipitation, and daily potential evapotranspiration at the study site from April to November in 2021–2023 **(a–c)**.

The cotton variety used in this study, J206-5, was approved by the China Crop Variety Approval Committee in 2016 and is suitable for spring planting in the early to mid-maturity cotton regions of the northwest inland area. The experiment was carried out from 2021 to 2023 in Shaya County (41°17’ N, 82°42’ E, 897 m above sea level), Xinjiang, Northwest China (**Figure 1**). The region is characterized by a warm temperate continental arid climate, with an average annual precipitation of 47.3 mm, evaporation of 2000.7 mm, sunshine duration of 3031.2 h, mean annual temperature of 10.7°C, maximum temperature of 30.9°C, minimum temperature of -13.7°C, and a frost-free period of 214 days. The daily mean temperature, precipitation, and potential evapotranspiration at the study site are depicted in **Figure 2**. Agricultural production in this region is entirely dependent on irrigation.

The experimental site featured sandy loam, with an average organic matter content of 9.8 g·kg^-1^, total nitrogen of 0.6 g·kg^-1^, alkali-hydrolyzed nitrogen of 39.5 mg·kg^-1^, available phosphorus of 18.1 mg⋅kg^-1^, available potassium of 111.9 mg·kg^-1^, and bulk density of 1.5 g·cm^-3^ in the topsoil. The soil pH was 8.3. The groundwater level at the experimental site was below 5 m, preventing any upward replenishment to the crop root zone.

### 2.2 Experimental design

**Figure 3:**
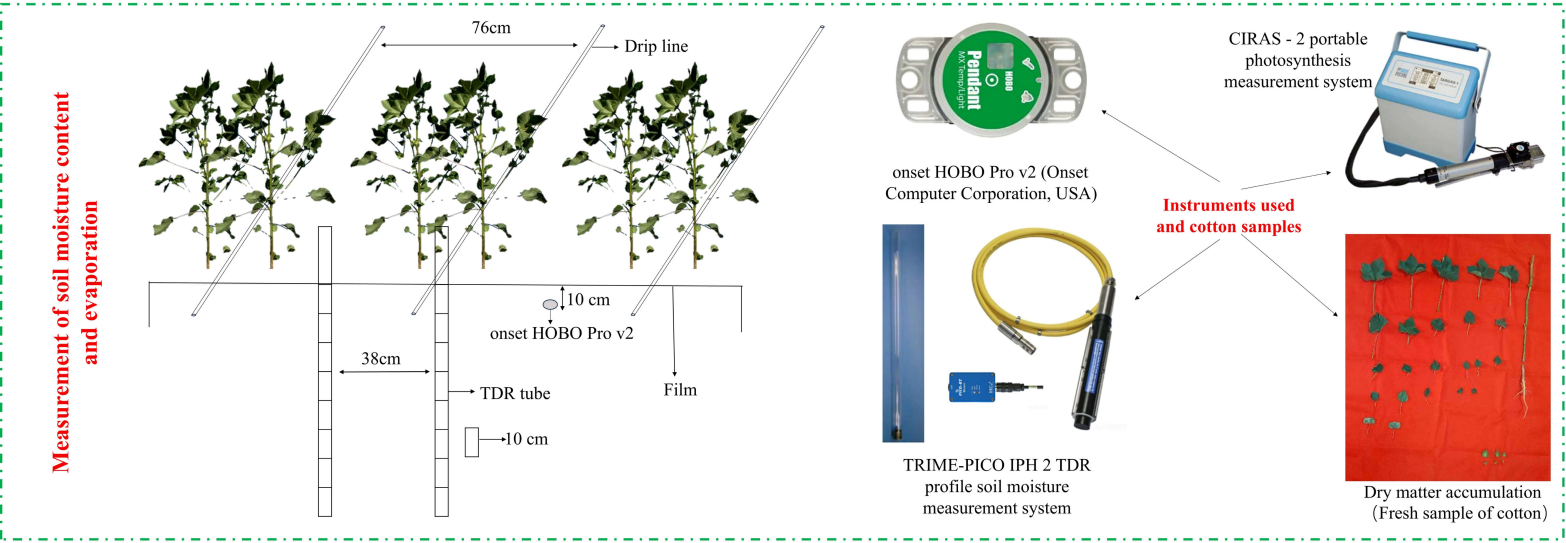
Cotton planting patterns and sampling and measuring sites.

A split-plot experimental design was adopted. The main plots were covered with film mulching, including one polyethylene mulch film (PE) and two biodegradable films (BEMFs: B1 and B2). The BEMFs, selected based on extensive experimental research, exhibit a stable degradation cycle and complete degradation within 100 days. The PE film is a conventional product widely used in the region. Detailed specifications of the films are provided in **Table 1**. Crop evapotranspiration (ETc) was calculated using the Penman-Monteith method recommended by the Food and Agriculture Organization (FAO). Three irrigation levels were established based on ETc: 63.6% ETc (W1, 315 mm), 81.8% ETc (W2, 405 mm), and 100% ETc (W3, 495 mm). Among these, W2 represents the conventional irrigation practice in the region. The experiment comprised nine treatments, each treatment was replicated three times. Each plot measured 9.5 m in length and 6.9 m in width, with a total area of 65.55 m². The planting configuration consisted of one film, three drip tubes, and six rows (**Figure 3**). The average plant spacing was 10.5 cm, and the row spacing was 38 cm, resulting in a theoretical planting density of 265,000 plants·ha^-1^. To minimize edge effects, the outer rows of each plot were designated as buffer zones, while the central row was used for data collection. The drip irrigation system featured emitters spaced at 25 cm intervals along the drip lines, which were spaced 76 cm apart. The emitter flow rate was 2.1 L·h^-1^. Water meters and control valves were installed for precise irrigation management. The water source was surface water storage in a reservoir. Irrigation schedules and volumes are detailed in **Table 2**. Field management practices followed standard local protocols.

**Table 1:** Mulching film data.

Type of mulching film	Treatments	Raw material	Width/m	Thickness/mm	Color	Induction period/d
Traditional polyethylene mulch	PE	polythene	2.05	0.01	transparent	No
Fully biodegradable mulch	B1	PBS and PBAT	2.05	0.01	transparent	100
Thermo-oxygen-biodegradable mulch	B2	polythene and biodegradation additives	2.05	0.01	transparent	100

PBS represents polybutylene succinate; PBA represents poly (butylene adipate)/terephthalate.

**Table 2:** Cotton growth process and irrigation time in the experimental area from 2021 – 2023.

Growth stage	Irrigation date			Irrigation quota (mm)		
	2021	2022	2023	W1	W2	W3
Sowing	May 18	April 10	April 10			
Budding stage	June 23	June 15	June 20	31.5	40.5	49.5
	June 30	June 22	June 27	31.5	40.5	49.5
Flowering stage	July 07	June 29	July 04	31.5	40.5	49.5
	July 14	July 06	July 12	31.5	40.5	49.5
	July 21	July 13	July 18	31.5	40.5	49.5
	July 28	July 20	July 24	31.5	40.5	49.5
Bolling stage	August 04	July 27	July 31	31.5	40.5	49.5
	August 11	August 03	August 07	31.5	40.5	49.5
	August 18	August 10	August 14	31.5	40.5	49.5
Boll opening stage	August 25	August 17	August 21	31.5	40.5	49.5
Total quota (mm)				315	405	495

### 2.3 Measurement items and methods

#### 2.3.1 Soil temperature

Soil temperature was measured using an Onset HOBO Pro v2 (Onset Computer Corporation, USA) automatic data logger. The sensor was placed at the center of the second row under the film in each plot, with the soil layer at a depth of 10 cm. Installation was completed within 24 h of cotton sowing, and the sensor recorded data automatically each hour.

#### 2.3.2 Soil moisture

Soil volumetric water content in the 0 - 80 cm soil layer of each plot was measured using the TRIME-PICO-IPH TDR (IMKO GmbH, Germany) throughout the cotton growing season. Measurements were taken at two points per plot: one in the wide row and one in the narrow row. The point in the wide row was located at the center of the second film, while the point in the narrow row was positioned directly below the drip emitter, corresponding to the wide row. Soil moisture was measured at 10 cm intervals with three repetitions per layer. Measurements were taken weekly, with additional measurements performed after irrigation and rainfall.

#### 2.3.3 Growth parameters

For each treatment, five consecutive representative plants were selected and marked at designated sampling points. On clear, sunny days between 12:00 and 16:00 Beijing time, during key cotton growth stages, the net photosynthetic rate (Pn) of functional leaves on the main stem (the fourth leaf from the top before topping and the third leaf from the top after topping) was measured using a portable photosynthesis system (CIRAS-2, Hansatech Company, King’s Lynn, UK) under natural light intensity (1600 μmol·m2·s^-1^).

A representative area with uniform growth was selected in the experimental field, and sampling plots were established. During the seedling, budding, full flowering, full boll, and boll opening stages, six representative cotton plants with uniform growth were chosen - three from the side row and three from the middle row. The plants were divided into leaves, stems, buds, bolls, flowers, and roots, then fixed at 105°C for 30 minutes and dried at 80°C until they reached a constant weight. After weighing the dry mass, the average value and distribution rate were calculated.

#### 2.3.4 Yield

Yield was measured when more than 80% of the cotton bolls had opened. To minimize errors, three uniformly growing and representative sample points were randomly selected in each replicate, with each sample point covering an area of 2.28 × 2.93 m (Shi et al., 2023). The number of plants and bolls was recorded, and the number of bolls per plant was calculated. Thirty cotton plants were randomly selected from each plots, and 30 bolls were collected from the upper, middle, and lower parts of these plants. After drying the bolls to a constant weight, the single boll weight and seed cotton yield were determined. Following ginning, the lint yield and lint percentage were measured.

The formula for calculating seed cotton yield (Shi et al., 2023):

Seed cotton yield = number of bolls per unit area  ×  single boll weight.

#### 2.3.5 Statistical analysis

Data analysis was performed using SPSS v.22.0 (SPSS Inc., Chicago, IL, USA). Significant differences among treatments were determined using the least significant difference (LSD) test at a significant differences level of P ≤ 0.05. Graphical representations of the results were generated using Origin Pro 2018 software (Origin Lab Corporation, Northampton, MA, USA).

## 3 Results

### 3.1 Seed cotton yield and yield gap.

**Table 3** presents the effects of PE and BEMF mulching on boll number per unit area, single boll weight, and seed cotton yield under different irrigation quotas. Compared to the highest seed cotton yield achieved under PE mulching, the yield under B1 and B2 mulching decreased by 17.35% and 17.02%, respectively, under the W1 irrigation quota, and by 7.51% and 7.52%, respectively, under the W2 irrigation quota (3-year average). In contrast, under W3 irrigation quota, the seed cotton yields under B1 and B2 mulching exceeded those under PE mulching by 16 kg·ha^-1^ and 82 kg·ha^-1^ in 2022, and by 28 kg·ha^-1^ and 12 kg·ha^-1^ in 2023, respectively. However, a continuous increase in the irrigation quota was not conducive to cotton yield formation of under PE mulching. Specifically, when PE mulching was applied, the boll number per unit area under the W3 irrigation quota was 1 boll·m^-2^ lower in 2021, and 8 bolls·m^-2^ lower in 2023, compared to that under the W2 irrigation quota.

**Table 3:** Cotton yield components under different irrigation quotas for biodegradable and traditional PE mulch.

Year	Treatments		Boll number (boll·m^-2^)	Single boll weight (g·per^-1^)	Seed cotton yield (kg·ha^-1^)	Yield gap (kg·ha^-1^)
2021	W1	PE	109 ± 7.23 a	5.60 ± 0.20 a	6096 ± 271 a	/
		B1	98 ± 1.39 b	5.48 ± 0.04 a	5388 ± 207 b	708 ± 150 b
		B2	105 ± 3.82 ab	5.16 ± 0.03 b	5356 ± 408 b	740 ± 110 a
	W2	PE	106 ± 3.52 a	5.55 ± 0.08 a	5874 ± 215 a	221 ± 52 a
		B1	105 ± 5.34 a	5.20 ± 0.08 b	5530 ± 390 b	565 ± 92 b
		B2	103 ± 3.91 a	5.35 ± 0.01 b	5450 ± 250 b	646 ± 179 a
	W3	PE	107 ± 1.02 b	5.07 ± 0.07 b	5872 ± 129 a	224 ± 64 b
		B1	110 ± 2.93 b	5.32 ± 0.02 a	5862 ± 148 a	234 ± 93 b
		B2	116 ± 0.95 a	5.06 ± 0.02 b	5434 ± 95 b	662 ± 99 a
2022	W1	PE	105 ± 3.73 a	5.91 ± 0.11 a	6201 ± 241 a	571 ± 113 a
		B1	99 ± 3.88 a	5.72 ± 0.05 b	5645 ± 280 b	1128 ± 132 a
		B2	98 ± 2.38 a	5.75 ± 0.08 b	5632 ± 229 b	1141 ± 85 b
	W2	PE	116 ± 1.95 a	5.86 ± 0.01 a	6772 ± 270 a	/
		B1	110 ± 1.92 b	5.75 ± 0.04 ab	6399 ± 271 b	427 ± 104 a
		B2	113 ± 5.51 ab	5.69 ± 0.08 b	6345 ± 284 b	373 ± 96 b
	W3	PE	108 ± 4.03 b	6.10 ± 0.08 a	6575 ± 210 b	198 ± 76 a
		B1	116 ± 3.34 a	5.88 ± 0.08 b	6789 ± 283 ab	-16 ± 127 b
		B2	112 ± 4.61 ab	6.11 ± 0.15 a	6854 ± 278 a	-82 ± 112 b
2023	W1	PE	102 ± 0.62 a	5.89 ± 0.05 a	6009 ± 156 a	926 ± 83 a
		B1	94 ± 2.42 b	5.65 ± 0.03 b	5403 ± 222 b	1649 ± 82 a
		B2	91 ± 2.71 b	5.91 ± 0.04 a	5286 ± 278 b	1532 ± 120 b
	W2	PE	117 ± 4.28 a	5.92 ± 0.07 b	6935 ± 272 a	/
		B1	108 ± 3.42 b	6.00 ± 0.05 a	6489 ± 269 b	481 ± 113 a
		B2	108 ± 4.22 b	5.99 ± 0.06 ab	6454 ± 292 b	447 ± 125 b
	W3	PE	109 ± 0.94 a	6.09 ± 0.06 a	6620 ± 287 b	315 ± 122 a
		B1	114 ± 2.19 a	6.13 ± 0.10 a	6963 ± 271 a	-28 ± 118 b
		B2	114 ± 4.66 a	6.10 ± 0.09 a	6948 ± 295 a	-12 ± 125 b
Source of variance						
Year (Y)			**	**	**	**
Irrigation quota (I)			**	**	**	**
Mulch (M)			**	**	**	ns
Y×I			**	**	**	**
Y×M			**	**	ns	**
I×M			**	**	**	**
Y×I×M			**	**	ns	**

Different letters within a column and experimental year represent significant differences at P ≤ 0.05. * and ** represent a significant difference at the 5 and 1% levels; ns represents no significant difference at the 5% level.

### 3.2 Soil temperature

**Figure 4:**
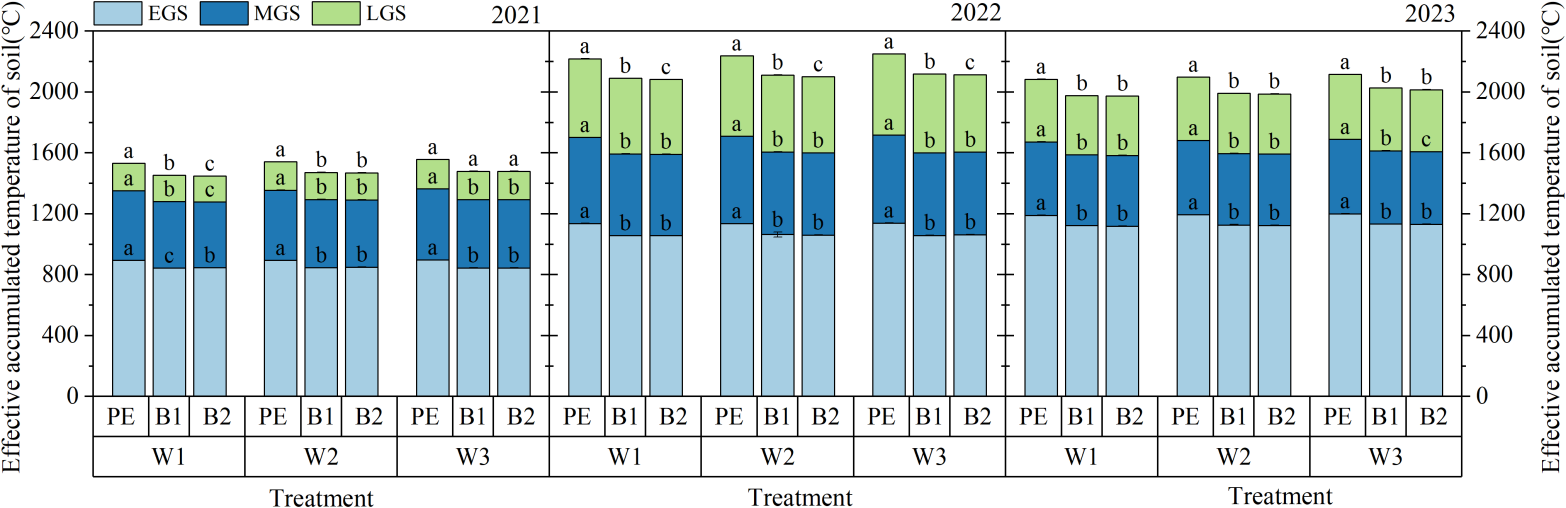
The accumulated amount of soil effective accumulated temperature at different growth stages of cotton under different mulching methods and irrigation quotas. W1 irrigation quota: 63.6% crop evapotranspiration (ETc) (315 mm); W2 irrigation quota: 81.8% ETc (405 mm); W3 irrigation quota: 100% ETc (495 mm); PE, polyethylene film; B1 and B2, biodegradable mulch film; EGS, Early growth stage; MGS, Middle growth stage; LGS, Late growth stage. Different letters within a column and experimental year represent significant differences at P ≤ 0.05.

**Figure 4** and **Table 4** illustrate the effects of PE, B1, and B2 mulching on the accumulation of under different irrigation quotas. The thermal insulation effect of B1 and B2 mulching was weaker than that of PE mulching, particularly during the early growth stages of cotton. In the early growth stage, the soil effective temperature accumulation under B1 and B2 mulching was 40.46 - 84.74°C lower than that under PE mulching. However, this difference gradually narrowed to 7.05 - 25.90°C during the middle and late growth stages. Increasing the irrigation quota improved soil temperature under plastic film mulching. Under B1 mulching, the soil effective temperature accumulation under the W3 irrigation quota was 15.52°C and 34.33°C higher than that under W2 and W1, respectively (3-year average).

**Table 4:** Effective soil temperature accumulation under different irrigation quotas for biodegradable and traditional PE mulch.

Year	Treatment		Soil effective temperature accumulation (°C)			
			WGS	EGS	MGS	LGS
Average value from 2021 - 2023	W1	PE	1945 ± 105c	1074 ± 45a	653 ± 17b	370 ± 49cd
	W1	B1	1840 ± 98gh	1007 ± 42b	634 ± 15ef	353 ± 48f
	W1	B2	1835 ± 98h	1007 ± 41b	632 ± 15f	350 ± 48f
	W2	PE	1960 ± 106b	1075 ± 46a	659 ± 17a	377 ± 50b
	W2	B1	1859 ± 98ef	1012 ± 43b	640 ± 14cd	360 ± 48e
	W2	B2	1853 ± 97fg	1011 ± 41b	637 ± 14de	358 ± 48e
	W3	PE	1975 ± 106a	1077 ± 46a	663 ± 17a	385 ± 50a
	W3	B1	1874 ± 100d	1011 ± 43b	644 ± 14c	372 ± 49c
	W3	B2	1870 ± 99de	1012 ± 43b	643 ± 14c	368 ± 48d
Source of variance						
Year (Y)			**	**	**	**
Irrigation quota (I)			**	*	**	**
Mulch (M)			**	**	**	**
Y×I			*	NS	**	**
Y×M			**	**	**	**
I×M			NS	NS	NS	**
Y×I×M			NS	NS	**	**

W1 irrigation quota: 63.6% crop evapotranspiration (ETc) (315 mm); W2 irrigation quota: 81.8% ETc (405 mm); W3 irrigation quota: 100% ETc (495 mm); PE, polyethylene mulch film; B1 and B2, biodegradable mulch film; WGS, Whole growth stage; EGS, Early growth stage; MGS, Middle growth stage; LGS, Late growth stage. Different letters within a column and experimental year represent significant differences at P ≤ 0.05. * and ** represent a significant difference at the 5 and 1% levels; NS represents no significant difference at the 5% level.

### 3.3 Soil water content

**Figure 5:**
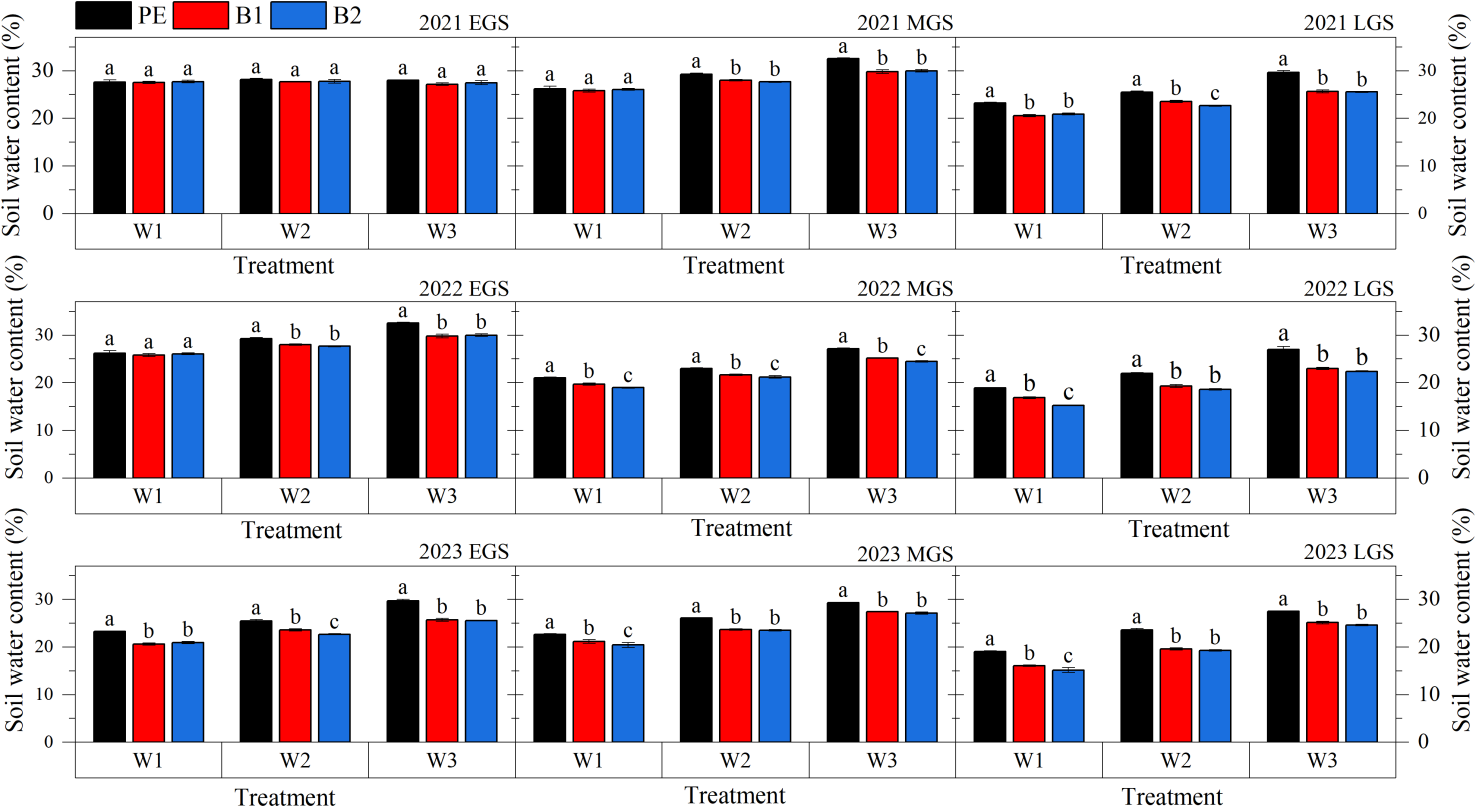
Average soil water content at different growth stages of cotton under different mulching methods and irrigation quotas. W1: 63.6% crop evapotranspiration (ETc) (315 mm); W2: 81.8% ETc (405 mm); W3: 100% ETc (495 mm); PE, polyethylene film; B1 and B2, biodegradable mulch film. EGS, Early growth stage; MGS, Middle growth stage; LGS, Late growth stage. Different letters within a column and experimental year represent significant differences at P ≤ 0.05.

**Figure 5** and **Table 5** illustrates the changes in the average SWC within the 0 - 80 cm soil layer throughout the cotton growth period. Under different plastic film treatments and irrigation quotas, the average SWC increased with higher irrigation quotas. Throughout the growth period, the PE treatment remained intact without degradation. In the 0 - 80 cm soil layer, the average SWC of under W3 quota was 20.17% and 10.72% higher than that under the W1 and W2 quotas, respectively. When covered with B1 mulching, the average SWC under the W1, W2, and W3 quotas was 5.75%, 6.75%, and 7.46% lower, respectively, compared to the same irrigation level under PE treatment. No significant differences were observed between B1 and B2 treatments. During the early growth stage of cotton, under the W1 quota, the average SWC under B1 and B2 mulching was 1.27% and 1.88% lower, respectively, than under PE mulching. Under the W2 and W3 quotas, the average SWC was 2.13 - 2.80% lower than that under PE mulching. In the middle and late stages growth stage, the degradable plastic film (B2) continuously degraded as the cotton growth period progressed, and its moisture retention effect gradually weakened. Under the W1, W2, and W3 quotas, the SWC under B2 mulching was 19.46%, 22.16% and 25.70%, respectively, which was 10.93%, 11.03% and 10.99% lower than that under PE mulching. No significant differences were observed between B1 and B2 treatments.

**Table 5:** Soil water content under different irrigation quotas for biodegradable and traditional PE mulch.

Year	Treatment		Soil water content (%)			
			WGS	EGS	MGS	LGS
Average value from 2021 — 2023	W1	PE	22.94 ± 0.58d	25.12 ± 0.64bc	23.28 ± 0.78e	20.42 ± 0.72d
	W1	B1	21.62 ± 0.63e	24.80 ± 0.68cd	22.22 ± 0.93f	17.84 ± 0.70e
	W1	B2	21.19 ± 0.77e	24.65 ± 0.76d	21.83 ± 1.09f	17.10 ± 0.97e
	W2	PE	25.10 ± 0.58c	25.50 ± 0.67ab	26.11 ± 0.91c	23.71 ± 0.52c
	W2	B1	23.41 ± 0.63d	24.96 ± 0.66cd	24.45 ± 0.94d	20.82 ± 0.70d
	W2	B2	23.07 ± 0.63d	24.89 ± 0.68cd	24.13 ± 0.95d	20.19 ± 0.63d
	W3	PE	27.80 ± 0.51a	25.65 ± 0.64a	29.68 ± 0.79a	28.06 ± 0.46a
	W3	B1	25.72 ± 0.45b	25.10 ± 0.55bc	27.47 ± 0.68b	24.61 ± 0.44b
	W3	B2	25.44 ± 0.53bc	24.93 ± 0.64cd	27.20 ± 0.81b	24.19 ± 0.47bc
Source of variance						
Year (Y)			**	**	**	**
Irrigation quota (I)			**	**	**	**
Mulch (M)			**	**	**	**
Y×I			**	*	**	**
Y×M			**	NS	*	*
I×M			*	NS	**	NS
Y×I×M			**	NS	*	*

W1 irrigation quota: 63.6% crop evapotranspiration (ETc) (315 mm); W2 irrigation quota: 81.8% ETc (405 mm); W3 irrigation quota: 100% ETc (495 mm); PE, polyethylene film; B1 and B2, biodegradable mulch film. WGS, Whole growth stage; EGS, Early growth stage; MGS, Middle growth stage; LGS, Late growth stage. Different letters within a column and experimental year represent significant differences at P < 0.05. * and ** represent a significant difference at the 5 and 1% levels; NS represents no significant difference at the 5% level.

### 3.4 Net photosynthetic rate

**Figure 6:**
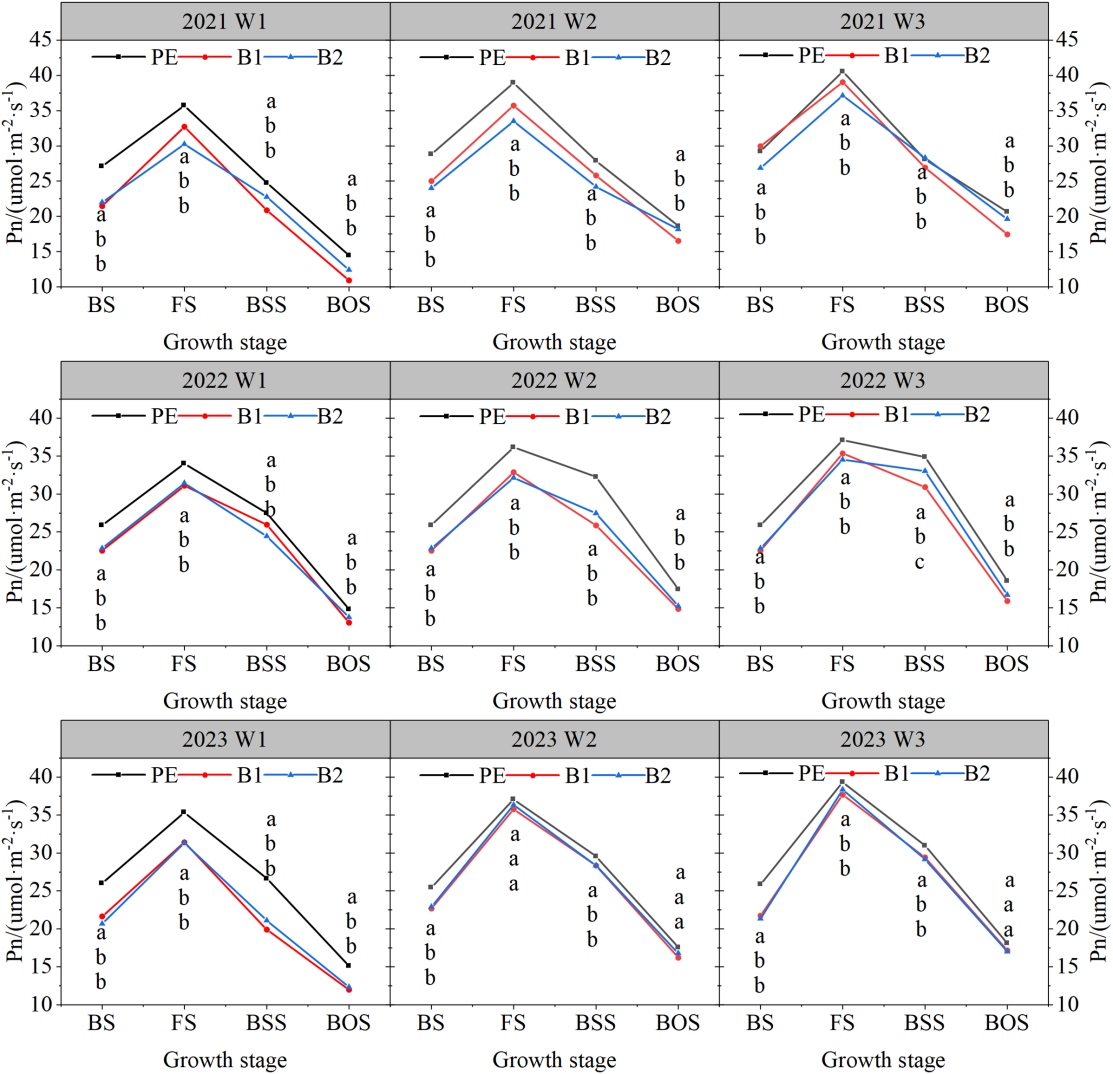
Net photosynthetic rate of main stem leaves of cotton at four main growth stages under different mulching methods and irrigation quotas. W1: 63.6% crop evapotranspiration (ETc) (315 mm); W2: 81.8% ETc (405 mm); W3: 100% ETc (495 mm); PE, polyethylene film; B1 and B2, biodegradable mulch film. SS, Seedling stage, 27 (2021)/38(2022)/51(2023) days after sowing; BS, Budding stage, 47(2021)/69 (2022)/75 (2023) days after sowing; FS, Flowering stage, 77 (2021)/83 (2022)/97 (2023) days after sowing; BSS, Bolling stage, 95/116/119 days after sowing; BOS, Boll opening stage, 127 (2021)/143 (2022)/148 (2023) days after sowing. Different letters within a column and experimental year represent significant differences at P< 0.05.

During the three experimental years, the trend in Pn throughout the cotton growth period was consistent across different BEMF treatments and irrigation quotas (**Figure 6**). Pn initially increased, peaked at the flowering stage, and then decreased. The Pn under PE treatment was significantly higher than that under B1 and B2 treatments (P ≤ 0.05). Specifically, the Pn under PE treatment was 10.29%, 6.71%, 10.78%, and 31.73% higher than that under the B1 treatment during four growth periods. Under the same mulching treatment, Pn increased with higher irrigation quotas. For example, at the flowering stage under B1 mulching the Pn under the W3 irrigation quota was 37.42 μmol·m^-2^·s^-1^, which was 9.71% and 4.87% higher than that under the W1 and W2 irrigation quotas, respectively (P ≤ 0.05 for both differences).

### 3.5 Dry matter accumulation

**Figure 7:**
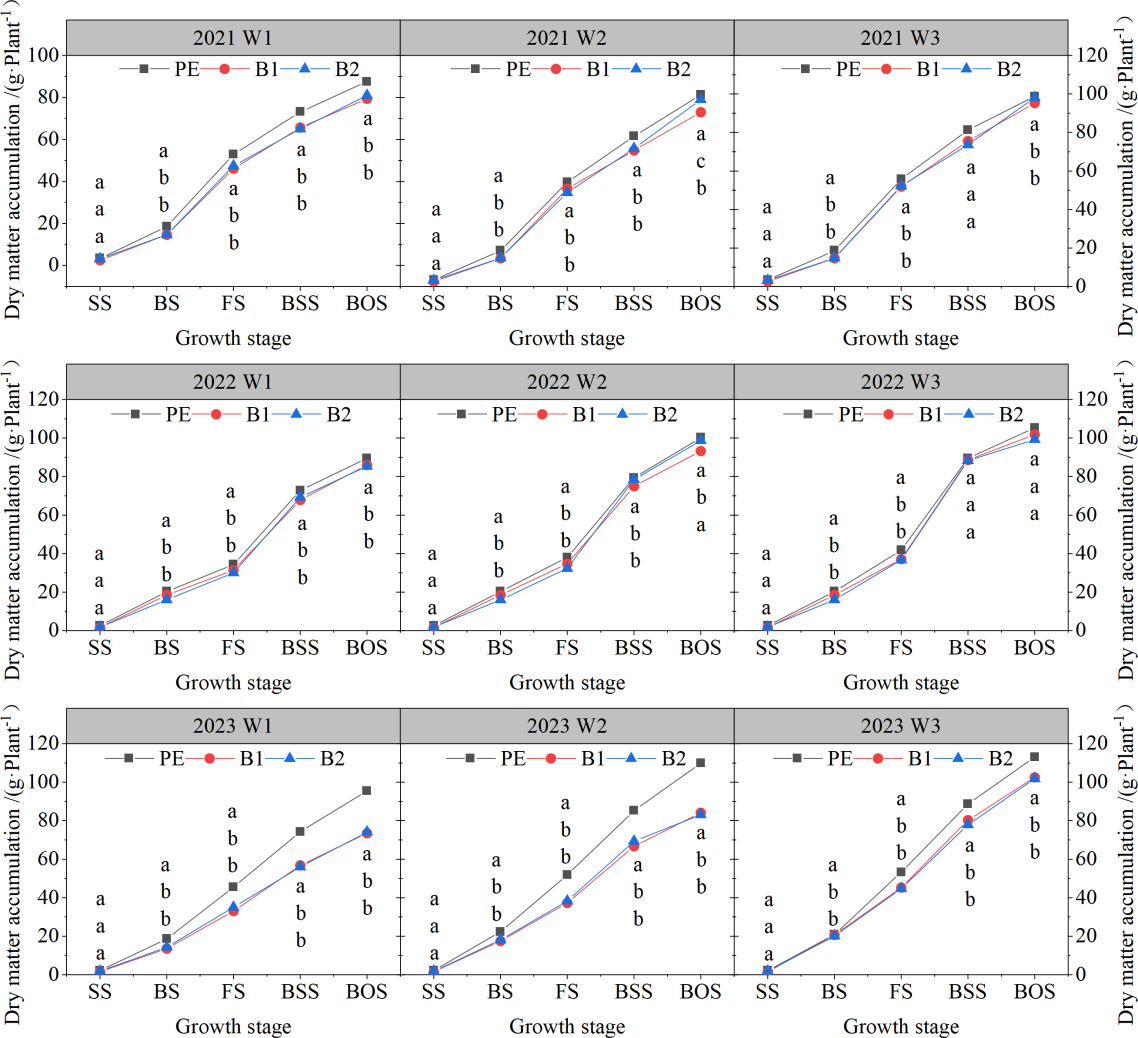
Dry matter accumulation of cotton under different mulching methods and irrigation quotas. W1: 63.6% crop evapotranspiration (ETc) (315 mm); W2: 81.8% ETc (405 mm); W3: 100% ETc (495 mm); PE, polyethylene film; B1 and B2, biodegradable mulch film. SS, Seedling stage, 27 (2021)/38(2022)/51(2023) days after sowing; BS, Budding stage, 47(2021)/69 (2022)/75 (2023) days after sowing; FS, Flowering stage, 77 (2021)/83 (2022)/97 (2023) days after sowing; BSS, Bolling stage, 95/116/119 days after sowing; BOS, Boll opening stage, 127 (2021)/143 (2022)/148 (2023) days after sowing. Different letters within a column and experimental year represent significant differences at P ≤ 0.05.

As shown in **Figure 7**, the dry matter accumulation (DM) of cotton under each treatment exhibited a gradually increasing trend through the growth period. When the irrigation quota was the same, the DM under PE mulching was significantly higher than that under B1 and B2 mulching. However, this difference gradually narrowed with increasing irrigation quota. For example, at the boll stage, the DM under PE mulching for the W1, W2, and W3 quotas was 73.63 g·plant^-1^, 80.86 g·plant^-1^ and 86.50 g·plant^-1^ respectively. These values were 10.22%, 9.45% and 7.03% higher, respectively, than the DM under B1 mulching at the same irrigation quotas. No significant differences were observed in DM between B1 and B2 mulching (P > 0.05).

### 3.6 Benefit analysis

The costs and benefits under different treatments are presented in **Table 6**. When the irrigation quota was the same, significant differences in production costs were observed among the plastic film mulching treatments. Compared to PE mulching, the production costs of B1 and B2 mulching increased by 630 CNY⋅ha^-1^ due to higher price of biodegradable films, and labor costs for weeding increased by 180 - 255 CNY·ha^-1^. However, these treatments reduced the costs associated with plastic film recovery and disposal by 600 CNY⋅ha^-1^. Under the same mulching conditions, the production cost increased by 0.2 CNY·ha^-1^ for every 1 m^3^·ha^-1^ increase in the irrigation quota. When covered with B1 and B2 mulching, the highest profits were achieved under the W3 irrigation quota, ranging from 18,702 - 39,443 CNY⋅ha^-1^. In 2021, the maximum profit under B1 and B2 mulching decreased by 2,289 CNY⋅ha^-1^ and 2,220 CNY⋅ha^-1^, respectively, compared to PE mulching. No significant differences in the maximum profit were observed in 2022 and 2023 when compared to PE mulching. Additionally, after mechanical recovery, 12.95 kg·ha^-1^ of plastic film fragments remained in the soil under PE mulching, whereas B1 and B2 mulching completely degraded, leaving no residue.

**Table 6:** Analysis of economic and ecological benefits of cotton fields under different mulching methods and irrigation quotas.

Treatments		Economic benefits (CNY⋅ha^-1^)									Ecological benefits (kg⋅ha^-1^)
		Agricultural capital investment			Labor	Film recycling and treating		Production value	Profit	Pv/P	Surface residual film quality
		Mulch	Water and electricity	Other		Film recycling	Film treating				
W1	PE	945	632	14205	2250	450	150	39622 ± 587 a	20991 ± 587 a	1.89 ± 0.08 b	12.95
W1	B1	1575	632	14205	2400	0	0	34814 ± 448 b	16002 ± 448 b	2.18 ± 0.10 a	0
W1	B2	1575	632	14205	2400	0	0	35023 ± 883 b	16212 ± 883 b	2.19 ± 0.23 a	0
W2	PE	945	812	14205	2325	450	150	38184 ± 465 a	19297 ± 465 a	1.98 ± 0.08 b	12.95
W2	B1	1575	812	14205	2475	0	0	35425 ± 844 b	16358 ± 844 b	2.19 ± 0.20 a	0
W2	B2	1575	812	14205	2475	0	0	35947 ± 541 b	16880 ± 541 b	2.14 ± 0.11 a	0
W3	PE	945	993	14205	2400	450	150	35321 ± 279 b	16178 ± 279 b	2.19 ± 0.06 a	12.95
W3	B1	1575	993	14205	2625	0	0	38100 ± 320 a	18702 ± 320 a	2.04 ± 0.05 b	0
W3	B2	1575	993	14205	2625	0	0	38169 ± 206 a	18771 ± 206 a	2.03 ± 0.03 b	0
W1	PE	945	632	14205	2250	450	150	43406 ± 563 a	24775 ± 563 a	1.76 ± 0.05 b	12.44
W1	B1	1575	632	14205	2400	0	0	39512 ± 654 b	20700 ± 654 b	1.92 ± 0.09 a	0
W1	B2	1575	632	14205	2400	0	0	39422 ± 535 b	20611 ± 535 b	1.92 ± 0.07 a	0
W2	PE	945	812	14205	2325	450	150	47406 ± 631 a	28519 ± 631 a	1.66 ± 0.04 b	12.44
W2	B1	1575	812	14205	2475	0	0	44416 ± 631 b	25349 ± 631 b	1.76 ± 0.06 a	0
W2	B2	1575	812	14205	2475	0	0	44792 ± 662 b	25725 ± 662 b	1.75 ± 0.06 a	0
W3	PE	945	993	14205	2400	450	150	46023 ± 649 b	26881 ± 649 b	1.72 ± 0.05 a	12.44
W3	B1	1575	993	14205	2625	0	0	47521 ± 659 a	28123 ± 659 ab	1.69 ± 0.05 ab	0
W3	B2	1575	993	14205	2625	0	0	47979 ± 490 a	28582 ± 490 a	1.68 ± 0.04 b	0
W1	PE	945	632	14205	2250	450	150	50778 ± 440 a	32146 ± 440 a	1.58 ± 0.02 b	13.19
W1	B1	1575	632	14205	2400	0	0	44670 ± 624 b	25858 ± 624 b	1.73 ± 0.05 a	0
W1	B2	1575	632	14205	2400	0	0	45654 ± 784 b	26843 ± 784 b	1.71 ± 0.06 a	0
W2	PE	945	812	14205	2325	450	150	58604 ± 766 a	39717 ± 766 a	1.48 ± 0.03 b	13.19
W2	B1	1575	812	14205	2475	0	0	54541 ± 757 b	35473 ± 757 b	1.54 ± 0.03 a	0
W2	B2	1575	812	14205	2475	0	0	54829 ± 823 b	35761 ± 823 b	1.53 ± 0.04 a	0
W3	PE	945	993	14205	2400	450	150	55942 ± 810 a	36799 ± 810 b	1.49 ± 0.04 a	13.19
W3	B1	1575	993	14205	2625	0	0	58840 ± 762 a	39443 ± 762 a	1.49 ± 0.03 a	0
W3	B2	1575	993	14205	2625	0	0	58709 ± 832 b	39311 ± 832 a	1.52 ± 0.03 a	0

Different letters within a column and experimental year represent significant differences at P< 0.05.

### 3.7 Correlation analysis

**Figure 8:**
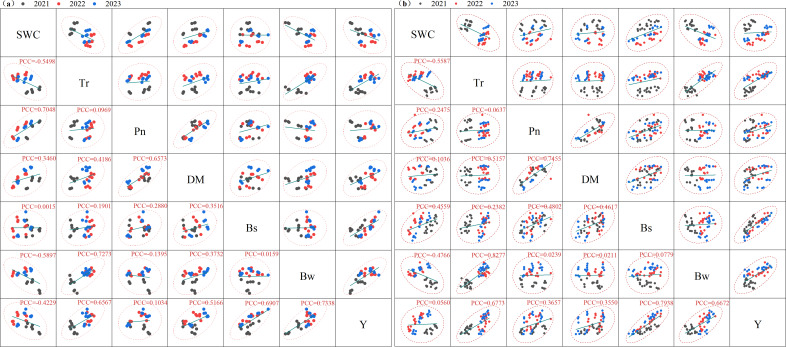
Correlation analysis of Pearson Correlation Coefficient (PCC) soil water content (SWC), soil temperature (Tr), cotton net photosynthetic rate (Pn), dry matter accumulation (DM), number of bolls per unit area (Bs), single boll weight (Bw), and seed cotton yield (Y) under the coverage of traditional polyethylene mulch **(a)** and biodegradable mulch **(b)**. W1: 63.6% crop evapotranspiration (ETc) (315 mm); W2: 81.8% ETc (405 mm); W3: 100% ETc (495 mm); PE, polyethylene film; B1 and B2, biodegradable mulch film.

**Figure 8** presents the correlation analysis of soil hydrothermal conditions, cotton photosynthetic performance, yield, and yield components under traditional PE mulching and BE mulching. Under PE mulching, the SWC showed a positively correlated with Pn and DM, with a highly significant correlation with Pn (P ≤ 0.01). However, SWC was negatively correlated with boll number per unit area (Bs), single boll weight (Bw), and seed cotton yield (Y), though these correlations were not significant (P > 0.05). Soil temperature (Tr) was positively correlated with Pn, DM, Bs, Bw, and Y, but only the correlation with Bw reached a significant level (P ≤  0.05). Under B1 and B2 mulching, both SWC and Tr were positively correlated with Pn, DM, Bs, and Y, but none of these correlations were significant (*P* > 0.05). In contrast, Tr showed a highly significantly positive correlation with Bw (P ≤ 0.01).

## 4 Discussion

### 4.1 Analysis of cotton yield and income earned under different mulching methods and irrigation quotas.

Crop growth and development are influenced by factors such as soil moisture and temperature. Within a certain range, the growth rate of crop is linearly correlated with changes in external factors. For example, higher soil water content and temperature are conducive to maize growth and development. Mulching can effectively reduce ground radiation heat loss, minimize soil water evaporation, improve soil temperature in the cultivated layer, alleviate soil salt accumulation, and enhance crop water absorption capacity, thereby increasing yield.

Pal and Mahajan (2017) research increased crop dry root yield by 24.3 - 49.7% compared with no mulching treatment. In our study, under the same irrigation quota, the seed cotton yield under BEMF was lower than that under traditional PEMF. This aligns with the findings of Zong et al. (2021) who reported that cotton yield under BEMF was significantly lower than under PEMF in terms of photosynthetic capacity and yield. The degradation and cracking of BEMF during the middle and late stages reduced its water and heat preservation performance, thereby decreasing cotton yield.

Under the W1 irrigation quota, the cotton yield under B1 and B2 mulching were 5,479 kg·ha^-1^ and 5,425 kg·ha^-1^, respectively, which were 16.06% and 16.89% lower than the highest yield under PE mulching. Under the W2 irrigation quota, the yields under B1 and B2 mulching were 6,139 kg·ha^-1^ and 6,083 kg·ha^-1^, respectively, 5.94% and 6.80% lower than under PE mulching. However, under the W3 irrigation quota, the yields under B1 and B2 mulching were 6,538 and 6,412 kg·ha^-1^, respectively, which B1 yielding 0.16% higher and B2 yielding 1.76% lower, than under PE mulching. These results indicate that increasing the irrigation quota compensated for water loss caused by BEMF, promoting cotton photosynthesis, photosynthetic products, and their translocation to reproductive organs, thereby increasing boll number, single boll weight, and overall yield. However, excessive irrigation can reduce the yield-increasing effect and even lower cotton yield. Yield formation is influenced not only by environmental and cultivation factors but also by the assimilation, transport, and utilization of photosynthetic products (Cao et al., 2024; Ma et al., 2024; Pettigrew and Gerik, 2007). We observed that the net photosynthetic rate (Pn) and dry matter accumulation (DM) under traditional PEMF were higher than under BEMF during key growth stages, demonstrating that soil moisture regulates photosynthetic efficiency. The lower water and heat preservation of BEMF affects cotton water absorption and photosynthesis. Increasing the irrigation quota under BEMF compensated for water demand, enhancing photosynthesis. For example, under B1 mulching, Pn increased by 10.07% and 17.80% under W2 and W3 irrigation quotas, respectively, compared to W1. Similarly, under B2 mulching, Pn increased by 8.92% and 17.79%, respectively. DM, the highest form of photosynthetic products, is crucial for yield formation (Pal and Mahajan, 2017; Shi et al., 2024; Wu et al., 2024).

Under traditional PEMF, DM in vegetative organs increased with irrigation quota, while DM in reproductive organs initially increased and then decreased. In contrast, under B1 and B2 mulching, increased irrigation promoted DM accumulation and its translocation to reproductive organs. This suggests that intact PEMF retains soil water, leading to vigorous vegetative growth but delayed and shortened reproductive growth, reducing yield. However, BEMF degradation increases evapotranspiration, preventing water enrichment and promoting reproductive growth.

Economic and ecological benefits varied with mulching treatments due to differences in mulch cost and soil hydrothermal conditions (Bo et al., 2022; Gao et al., 2022; Meng et al., 2022). Under W2 irrigation, the highest income from PE mulching was 29,178 CNY·ha⁻¹, while under W3 irrigation, incomes under B1 and B2 mulching were 28,576 CNY·ha^-1^ and 28,888 CNY·ha^-1^, respectively, with no significant differences among treatments. However, under W1 and W2 irrigation, incomes under B1 and B2 mulching were significantly lower than under PEMF, consistent with Sun et al. (2018) and Bo et al. (2022). This is attributed to poorer hydrothermal conditions under B1 and B2, reducing yield by 5.95 - 11.03%, and higher mulch and labor costs (180 - 255 CNY·ha^-1^). Under W3 irrigation, high soil water content reduced oxygen concentration, inhibiting root growth and yield by 171 kg·ha^-1^ compared to W2. Additionally, increased irrigation raised water and electricity costs, reducing economic benefits under PE mulching by 2,558 CNY·ha^-1^. In contrast, increased irrigation under B1 and B2 mulching compensated for soil water loss, meeting cotton water demand and increasing yield, outweighing additional costs. Comprehensive evaluation indicates that PE mulching under W2 irrigation, B1 and B2 mulching under W3 irrigation provide the highest economic and ecological benefits, with no significant differences among treatments. These findings align with Bo et al. (2022) and Meng et al. (2022) and demonstrate that increasing irrigation can enable BEMF to match PEMF performance while avoiding plastic residue pollution.

### 4.2 The effects of different mulching methods on soil moisture and temperature and their response to irrigation quotas

As a simple water-saving measure, mulching has been widely adopted in agricultural production (Gu et al., 2016; Sapakhova et al., 2024). It effectively inhibits soil evaporation, reduces ineffective water consumption, and enhances water use efficiency (Fuchs and Hadas, 2011; Huang et al., 2023). In regions such as the Xinjiang oasis, where cotton cultivation is heavily reliant on mulching due to low annual precipitation (<200 mm) and high evaporation (>2000 mm), mulching is indispensable (Wang et al., 2021). However, the drawbacks of traditional polyethylene film mulching (PEMF) are becoming increasingly apparent. The accumulation of residual plastic film impedes water infiltration, disrupts soil moisture distribution, reduces soil porosity and aeration, and ultimately affects crop yield (Dewi et al., 2024). Consequently, biodegradable film mulching (BEMF) has garnered attention as a sustainable alternative to PEMF in oasis cotton regions.

Our research indicates that soil moisture content under BEMF is 12.19 - 18.61% lower than under PEMF, consistent with the findings of Yin et al. (2019). This reduction is attributed to the gradual degradation of BEMF, which leads to the formation and expansion of cracks on the film surface, diminishing its ability to retain soil moisture (Liu et al., 2022). As a result, soil water evaporation increases, leading to a decline in soil moisture content. Prolonged use of BEMF may exacerbate soil moisture depletion, potentially causing irreversible soil desiccation. However, our study demonstrates that appropriately increasing irrigation quotas can compensate for soil moisture loss, mitigating the risk of soil quality degradation associated with long-term BEMF use. With increased irrigation, soil moisture content under B1 and B2 mulching increased by 16.70 - 32.52% and 18.07 - 35.12%, respectively. The improved soil moisture promotes cotton growth and canopy development, further reducing soil water evaporation. Numerous studies (Braunack et al., 2015; Di Miceli et al., 2024; Yin et al., 2019) have demonstrated that mulching can effectively increase soil temperature, thereby promoting crop growth and development and ultimately enhancing yield. However, the impact on soil temperature varies depending on the mulching material used. Most studies suggest that the warming effect of BEMF is less pronounced than that of traditional PEMF. Our finding support this observation. Specifically, compared to PE, the warming effect of B1 and B2 was weaker, with the effective accumulated soil temperature throughout the cotton growth period reduced by 100 - 111°C.

These findings primarily reflect the fact that t traditional PEMF is tightly constructed, blocking water vapor exchange between the soil surface and atmosphere. During the degradation of BEMF, the area for water vapor exchange between the soil and the atmosphere increases (Liu et al., 2022) Consequently, evaporation extracts heat from the soil, lowering its temperature, and the reduced soil moisture content results in poorer thermal conductivity, causing the soil temperature to rise more slowly. Therefore, the insulating effect of the soil under BEMF is weaker than that of traditional mulch films. As the canopy gradually closes, a closed space forms between the canopy and the ground, creating a water vapor cycle within the cotton field’s canopy, which further undermines the insulating effect of the mulch. This phenomenon explains why, despite the degradation and cracking of BEMF during this stage—resulting in a gradually closes, a closed space forms between the canopy and ground, creating a water vapor cycle within the cotton field’s canopy, which further diminishes the insulating effect of the mulch. This phenomenon explains why, despite the degradation and cracking of BEMF during this stage—resulting in a gradual reduction in coverage—the temperature difference in the soil remains smaller compared to traditional PE. Additionally, increasing the irrigation quota elevated the soil temperature and narrowed the gap in effective accumulated soil temperature between PE and B1 and B2 mulching. An increase in the irrigation quota promoted photosynthetic production and increased the leaf area index (LAI) of cotton, further enhancing canopy closure and weakening the warming function of the mulch. Moreover, increasing the irrigation quota increased the SWC, thereby increasing the soil’s heat capacity and decelerating the loss of soil temperature.

### 4.3 Perspectives and limitations of this study

While PEMF has significantly increased crop yields, its long-term use has led to the accumulation of residual plastic in the soil. This accumulation reduces soil water permeability, accelerates organic carbon decomposition, and decreases soil fertility, posing challenges to the sustainable development of agricultural systems (Sun et al., 2020). We argue that enhancing crop productivity should not come at the expense of soil quality degradation, as the long-term functionality of agricultural ecosystems must be preserved. BEMF, which degrades completely into CO_2_ and H_2_O, offers a promising alternative to mitigate PEMF residue accumulation and associated environmental pollution (Serrano-Ruiz et al., 2021; Sintim et al., 2019). The results of our three-year study demonstrate that BEMF can fully degrade, eliminating the risks posed by PE residues. However, this degradation may lead to losses in soil moisture and temperature, negatively impacting cotton yield formation. Prolonged use of BEMF could significantly deplete soil moisture in the plow layer, potentially degrading soil quality. Our research suggests that appropriately increasing irrigation quotas during BEMF application can help mitigate these issues. Unfortunately, our study did not address whether adjustments in fertilization strategies are necessary to meet the new growth demands of cotton under increased irrigation quotas. Additionally, while BEMF degradation can provide a carbon source for specific microorganisms, potentially enhancing soil microbial diversity (Huang et al., 2022; Song et al., 2024), it remains unclear whether this process could lead to significant nitrogen consumption, resulting in an imbalance in the soil carbon-to-nitrogen (C:N) ratio. Therefore, future research should focus on optimizing irrigation and fertilization strategies for the W2 and W3 irrigation quotas to elucidate the long-term impacts of BEMF on soil quality.

## 5 Conclusion

In this study, although biodegradable mulch film effectively mitigates the issue of plastic pollution associated with polyethylene mulch film, it was observed that soil moisture and effective temperature under biodegradable mulching film decreased by 2.82 - 9.42% and 100 - 111°C, respectively, leading to a reduction in cotton yield by 7.51 - 17.35%. The economic benefits associated with biodegradable mulching film are significantly lower than those of traditional polyethylene mulch film, and prolonged coverage may also result in the depletion of moisture in the soil layer. Increasing the irrigation quota can help offset the negative impacts of biodegradable mulching film. Specifically, compared to W1, raising the irrigation quota can increase soil moisture content under biodegradable mulch film by 16.70 - 35.12% and raise effective soil temperature by 18 - 35°C. The improvements in soil moisture and temperature subsequently lead to increases in the net photosynthetic rate and dry matter accumulation of cotton under biodegradable mulching film by 13.51 - 22.39% and 12.31 - 25.39%, respectively, resulting in an increase in cotton yield by 12.06 - 19.34%. Over the course of three years, the results indicate that when the irrigation quota increases by approximately 18%, cotton yield and economic benefits under biodegradable mulching film can match or exceed those observed under polyethylene mulching film, with no residual materials left in the cotton fields. Considering the long-term economic and ecological benefits to the agricultural ecosystem, we recommend that, under feasible conditions, the irrigation quota for BEMF application should be increases by approximately 18%.

## Data Availability

The original contributions presented in the study are included in the article/supplementary material. Further inquiries can be directed to the corresponding authors.
